# Supplementary material for: Inter-laboratory variability in cytomegalovirus DNA quantification: implications for standardization and clinical monitoring
Source: J Clin Microbiol. 2025 Jun 5;63(7):e01911-24. doi: 10.1128/jcm.01911-24 (PMC12239724; doi:10.1128/jcm.01911-24)

**Supplementary table**: Characteristics of the different workflows used at each site


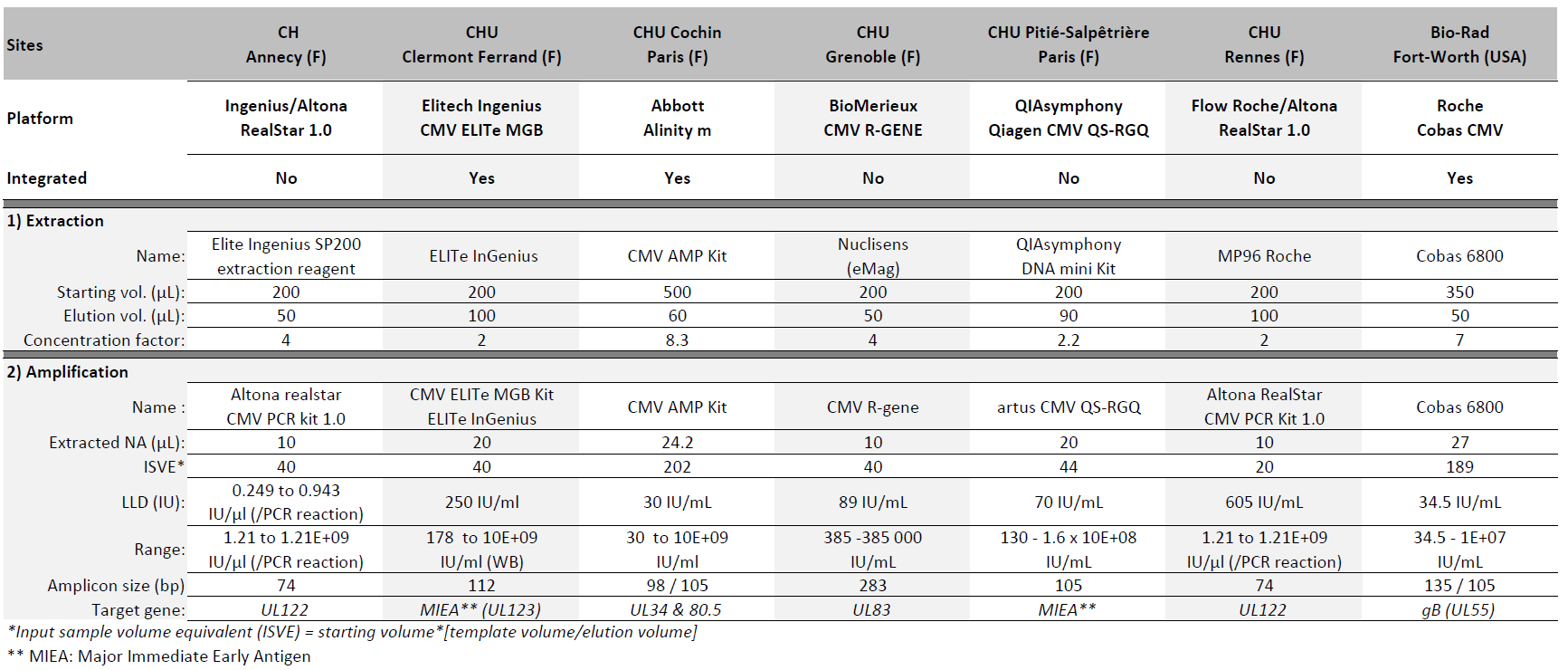


**Supplementary figure 1: Correlation between the nominal and measured values of the EDX standard when quantified by different systems.**

Closed squares: Alinity; Closed triangles: Ingenius; Closed circles: Ingenius-Altona; Open triangles: Nuclisens-Rgene; Stars: Qiagen-Artus; Open circles: Cobas; Open squares: Roche-Altona


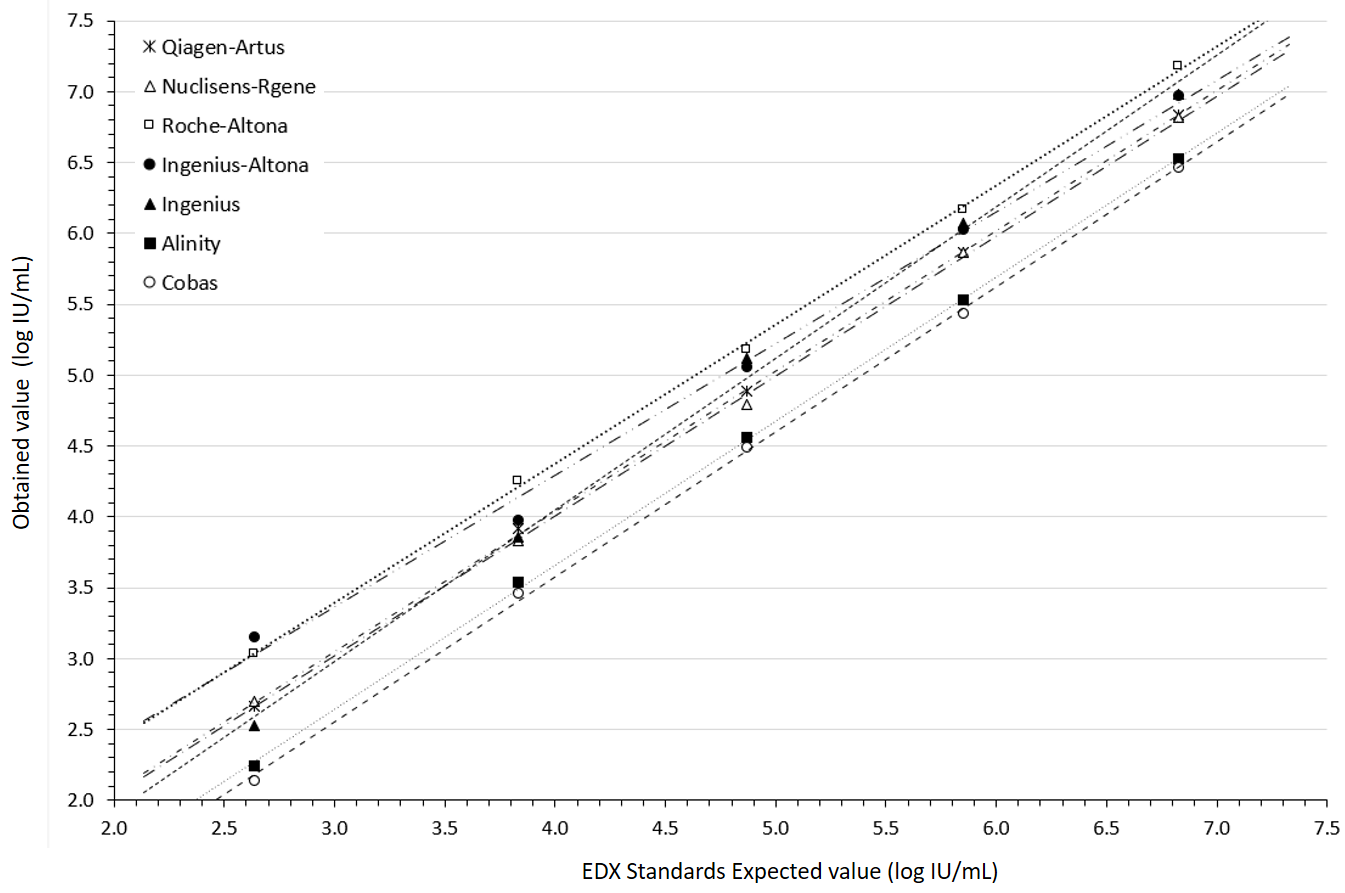


**Supplementary figure 2: Mean VL measured by all the systems combined for each panel member. Error bars represent the standard deviation.**

Closed circle: In-kit calibration; Open square: After recalibration against the WHO standard.


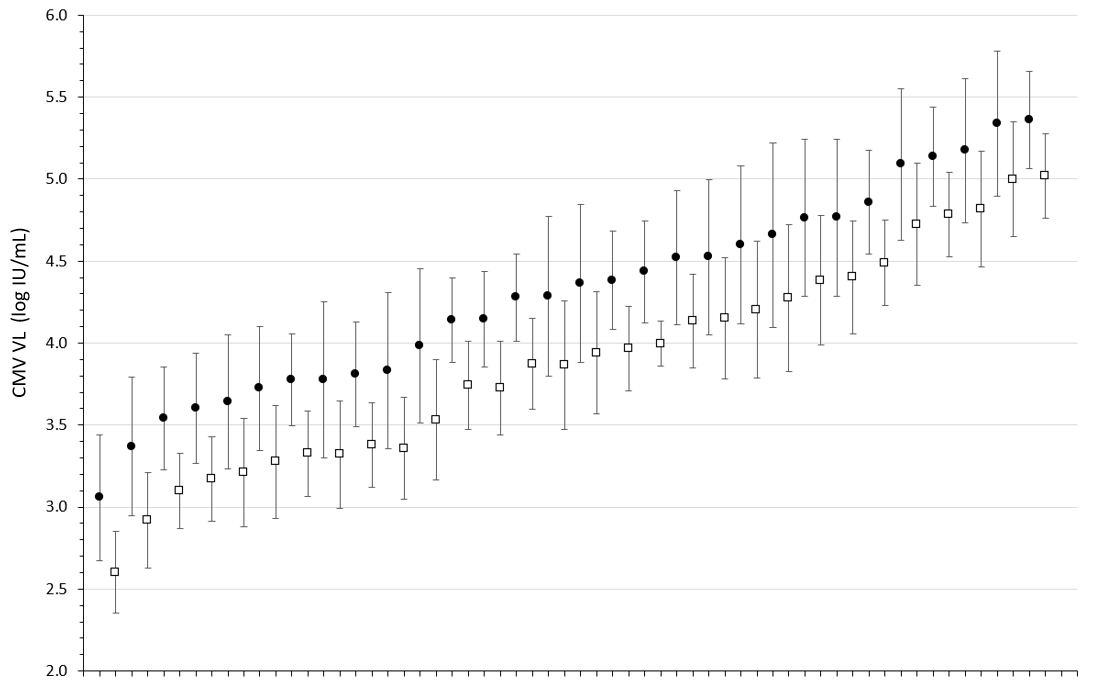

Supplement: Supplemental tables — Comprehensive table of the techniques and supplemental graphs. [file jcm.01911-24-s0001.docx]
